# Dopaminergic Neuronal Differentiation from the Forebrain-Derived Human Neural Stem Cells Induced in Cultures by Using a Combination of BMP-7 and Pramipexole with Growth Factors

**DOI:** 10.3389/fncir.2016.00029

**Published:** 2016-04-20

**Authors:** HongNa Yang, Jing Wang, Feng Wang, XiaoDun Liu, Heng Chen, WeiMing Duan, TingYu Qu

**Affiliations:** ^1^Department of Critical-Care Medicine, Qilu Hospital of Shandong University, Shandong UniversityJinan, China; ^2^Department of Psychiatry, College of Medicine, University of Illinois at ChicagoChicago, IL, USA; ^3^Department of Research and Development, Cell and Tissue Bank of Shandong ProvinceJinan, China; ^4^Department of Anatomy, Capital Medical UniversityBeijing, China

**Keywords:** dopaminergic neurons, TH, forebrain-derived human neural stem cells, PRX, BMP-7, dopamine release

## Abstract

Transplantation of dopaminergic (DA) neurons is considered to be the most promising therapeutic strategy for replacing degenerated dopamine cells in the midbrain of Parkinson's disease (PD), thereby restoring normal neural circuit function and slow clinical progression of the disease. Human neural stem cells (hNSCs) derived from fetal forebrain are thought to be the important cell sources for producing DA neurons because of their multipotency for differentiation and long-term expansion property in cultures. However, low DA differentiation of the forebrain-derived hNSCs limited their therapeutic potential in PD. In the current study, we explored a combined application of Pramipexole (PRX), bone morphogenetic proteins 7 (BMP-7), and growth factors, including acidic fibroblast factor (aFGF), forskolin, and phorbol-12-myristae-13-acetate (TPA), to induce differentiation of forebrain-derived hNSCs toward DA neurons in cultures. We found that DA neuron-associated genes, including Nurr1, Neurogenin2 (Ngn2), and tyrosine hydroxylase (TH) were significantly increased after 24 h of differentiation by RT-PCR analysis (*p* < 0.01). Fluorescent examination showed that about 25% of cells became TH-positive neurons at 24 h, about 5% of cells became VMAT2 (vascular monoamine transporter 2)-positive neurons, and less than 5% of cells became DAT (dopamine transporter)-positive neurons at 72 h following differentiation in cultures. Importantly, these TH-, VMAT2-, and DAT-expressing neurons were able to release dopamine into cultures under both of the basal and evoked conditions. Dopamine levels released by DA neurons produced using our protocol were significantly higher compared to the control groups (*P* < 0.01), as examined by ELISA. Our results demonstrated that the combination of PRX, BMP-7, and growth factors was able to greatly promote differentiation of the forebrain-derived hNSCs into DA-releasing neurons.

## Introduction

Parkinson disease (PD) as one of the most common neurodegenerative diseases is characterized by marked depletion of dopamine caused by dopaminergic neurons loss or degeneration in substantia nigra (SN) of the midbrain. To date, PD is still considered to be incurable and irreversible to the disabilities although the levodopa replacement therapy significantly remits the symptoms (Suzuki et al., 2015). Cell replacement therapy for PD, especially dopaminergic (DA) neurons, is considered to be the most promising candidate for restoring nigrostriatal DA transmission in the treatment of PD, Embryonic stem cells (ESCs) have been successfully induced to differentiate into DA neurons *in vitro* using various protocols (Kirkeby et al., 2012; Yang et al., 2014; Lim et al., 2015). However, many problems in ESCs, including their propensity to form teratomas and ethical issues, limit their clinical uses (Christophersen et al., 2006; Knoepfler, 2009). Although specific DA neuron-associated gene overexpression in midbrain-derived NSCs resulted from experimental gene transfection technology could produce DA neurons *in vitro* (Andersson et al., 2007), there is a safety concern for clinical uses of such gene-transfected cells. Midbrain-derived hNSCs may be more intended to differentiate into DA neurons, however, clinical application has been hindered due to the lack of sufficient midbrain tissues. In addition, midbrain-derived hNSCs will lose their proliferative property and multipotency for differentiation in long term cultures (Christophersen et al., 2006). Human neural stem cells (hNSCs) have been successfully isolated from fetal forebrains and these forebrain-derived hNSCs can be expanded in cultures for more than a year without losing their multipotency to differentiate into neurons and glial cells (Christophersen et al., 2006). Thus, forebrain-derived hNSCs can serve as suitable cell sources to provide sufficient number of candidate cells and to differentiate into DA neurons for transplantation uses in PD. More importantly, forebrain-derived hNSCs do not intend to form tumors when used for transplantation (Daadi et al., 2012). However, forebrain-derived hNSCs appear to hardly differentiate into functional DA neurons, lacking the capacity to release dopamine, compared to midbrain-derived hNSCs, which limited their therapeutic application in PD.

Nurr1, known as a member of the nuclear receptor family, is not only essential for the development of mesencephalic DA neurons but also a regulatory factor of differentiation, migration, and maturation of mesencephalic DA neurons (Li et al., 2011). Nurr1 is also involved in establishing the DA neurotransmitter identity of DA neurons in cultured cells, including ES cells, primary neural precursors derived from forebrain cortex, midbrain, and developing striatum. However, over-expression of Nurr1 alone is not sufficient to induce the expression of additional markers of DA neurons except for tyrosine hydroxylase (TH) (Andersson et al., 2007). Neurogenin2 (Ngn2) is proved to be critical for DA neuron differentiation of mesencephalic progenitors (Thompson et al., 2006). Over-expression of Ngn2 alone in midbrain-derived progenitors is not sufficient either to result in DA neurons except for neuronal maturation (Andersson et al., 2007). However, it has been reported that progenitors derived from fetal mouse ventral midbrain can further develop into neuronal cells expressing not only TH but also additional DA neuron markers, such as vascular monoamine transporter (VMAT2), by over-expression of gene Nurr1 and Ngn2 (Andersson et al., 2007). Although TH-expressing neurons can be induced from forebrain progenitors using growth factors, the percentage of these TH-expressing neurons is low (4–10%) and other DA neuron markers cannot be detected in these differentiated cells (Christophersen et al., 2006).

Pramipexole (PRX), as a non-ergot DA D2/D3 receptor agonist, is attracting more and more attention because of its effect on the treatment of early as well as advanced PD. *In vivo* experiments showed that PRX can can promote DA neurogenesis and increase motor activity in a PD animal model (Winner et al., 2009). *In vitro* experiments demonstrated that PRX can significantly induce the expression of DA neuron-associated genes [Nurr1, DAT (dopamine transporter), and VMAT2] in SH-SY5Y cells (Pan et al., 2005). Bone morphogenetic proteins 7 (BMP-7), as one of the transforming growth factor β (TGF-β) family members, has been proven to increase the number of TH-positive cells and dopamine uptake of rat mesencephalic cells *in vitro* (Lee et al., 2003). Its application can also significantly decrease DA neurons loss caused by 6-OHDA (6-hydroxydopamine) *in vivo* (Harvey et al., 2004). In addition, BMP-7 is able to up-regulate and maintain the expression of Ngn2 (Segklia et al., 2012).

Because BMP7 and PRX can upregulate the expression of DA neuron-associated key genes Nurr1 and Ngn2, and growth factors including acidic fibroblast factor (aFGF), forskolin, and phorbol-12-myristae-13-acetate (TPA), have been confirmed in previous studies to enhance the expression of TH in forebrain-derived hNSCs (Christophersen et al., 2006), we explored the possibility in the current studies to promote the differentiation of forebrain-derived hNSCs into DA-producing neurons by a combined application of PRX and BMP-7 with these growth factors.

## Methods and materials

### Culture of hNSCs (human neural stem cells)

hNSCs were purchased from Lonza (Walkersville, MD, USA). The detailed methods for the growth and maintenance of undifferentiated hNSCs were conducted according to the protocol we previously published (Ma et al., 2011). In brief, hNSCs were cultured in T-75 flask containing 15 ml complete culture medium including DMEM/F12 (Invitrogen, CA, USA), human recombinant epidermal growth factor (EGF; 20 ng/ml) and basic fibroblast growth factor (bFGF; 20 ng/ml) (R&D Systems, Minneapolis, MN, USA), B27 (serum-free medium supplements formulated to provide optimal growth condition for NSC expansion, 1:50; Invitrogen), heparin (5 μg/ml; Sigma, St Louis, MO, USA), 2 mM L-glutamine, and an antibiotic–antimycotic mixture (1: 100; Invitrogen) at 37°C in a 5% CO2 humidified incubation chamber (Fisher, Pittsburgh, PA, USA). 7.5 ml fresh complete culture medium was changed every 3 days. Every 1–2 week, we cut the larger neuro-spheroids into small spheroids under the observation of the microscope (Olympus, Japan).

### Induction of dopaminergic differentiation

hNSC spheres were mechanically dissociated into small spheres, part of which were plated on 12 mm (diameter) glass coverslips coated with poly-l-lysine in 1 ml complete culture medium supplemented with 1% fetal bovine serum (FBS) for promoting cell attaching to the coverslips. Other spheres were further prepared into single cells using accutase (Chemicon) and 1 × 10^6^ of single cells were plated in the 50 mm (diameter) tissues culture dishes in 4 ml complete culture medium supplemented with 1% FBS overnight for PCR and dopamine release analysis. On the second day, the medium were completely discharged and replaced with the same volume of differentiated medium, which included B27 medium supplemented with 1% FBS, BMP-7 (bone morphogenetic proteins, Peprotech, 50 ng/ml), pramipexole (PRX, Sigma, 10 ìm), and growth factors, including acidic fibroblast factors (aFGF, Sigma, 100 ng/ml), forskolin (Sigma, 25 ìm), and phorbol-12-myristae-13-acetate (TPA, Sigma, 100 nm). The controls were divided into two groups: one was cultured in B27 medium supplemented with 1% FBS and growth factors, the other was cultured in B27 medium supplemented with 1% FBS.

### RT-PCR

For PCR analysis, the cells were cultured *in vitro* for 24 h. Total RNA was extracted with Trizol (Invitrogen) following the manufacturer's protocol. One microgram of total RNA was used for reverse transcription using ImProm-II™ Reverse Transcription System (Promega) in a total volume of 20 μl. cDNA from all samples was synthesized at the same time using the same master mix to avoid variations. All PCRs were performed by using Quick-Load^®;^ Taq Master Mix (NEB) with 2 μl cDNA and final concentration of primer at 0.5 μM. The final reaction volume of PCR was 20 μl. All reactions were performed in triplicate. β-actin was used as internal control for equal loading of cDNA. PCR products were separated on 1.5% agarose gel, and quantified by densitometric analysis with quantity one system (Bio-Rad, USA). Data was from four independent experiments. The primers used in the research were listed as follows: β-actin: F (forward): 5′-GAA GTC CCT TGC CAT CCT AAA-3′; R (Reverse): 5′-GTC TCA AGT CAG TGT ACA GGT AAG-3′; Nurr1: F: 5′-GAA CTG CAC TTG GGC AGA GTT G-3′; R: 5′-GGT GGA CAG TGT CGT AAT TC-3′; Ngn2: F: 5′-GGT CTG GTA CAC GAT TGC AAA C-3′; R: 5′-GCT GTT GGT GCA ACT CCA CGT-3′; TH: F: 5′-TGT CCA CGC TGT ACT GGT TC-3′; R: 5′-AGC TCC TGA GCT TGT CCT TG-3′.

### Immunocytochemistry

After induction *in vitro* for 24 and 72 h, respectively, the differentiated cells were fixed in 4% paraformaldehyde (PFA) in phosphate buffered saline (PBS) for 20 min at room temperature (RT). The cells were washed three times with PBS, followed by overnight incubation with primary antibodies diluted in PBS containing 5% normal donkey serum (Jackson Immunoresearch Laboratories), 0.1% Triton X-100 at 4°C in a humidified chamber. On the second day, the cells were washed three times with PBS and incubated for 1 h at room temperature in the dark with secondary antibodies diluted in PBS. After washing three times with PBS, the cells were cover slipped with medium for fluorescence with DAPI (Vector Labs) and viewed by fluorescence microscopy (Olympus, Japan). The primary antibodies used were: mouse anti-β-tubulin III (Sigma 1:400); rabbit anti-TH (PelFreez 1:200); rabbit anti-DAT (Millipore 1:400), and rabbit anti-VMAT2 (Santa cruz 1:100). The secondary antibodies used in these experiments were: anti-mouse TRITC and anti-rabbit FITC (all from Jackson Immunoresearch Laboratories). In order to quantify the percentage of TH-, DAT-, or VMAT2-immunopositive cells out of the total cell population, cells were counted at 200 × magnification, three fields were randomly chosen in each culture and the percentage of TH-, DAT-, or VMAT2-immunopositive cells was calculated with respect to the total number of cells indicated by DAPI-positive nuclei. Data are from four independent experiments. On average, 400 cells were examined in each experiment.

### Dopamine release

Dopamine release was quantified using ELISA kits (Dopamine Research EIA, LaborDiagnostika Nord GmbH & Co. KG, German) analyses after 3 days of differentiation *in vitro*. Every group was divided into two subgroups. Subgroup1 was incubated in 1.5 ml HBSS for 15 min to test basal dopamine release. Subgroup2 was incubated in 1.5 ml HBSS plus 56 mM KCl for 15 min to measure evoked dopamine release. All samples were stabilized immediately with orthophosphoric acid (7.5%)/metabisulfate (0.22 mg/ml) and stored at −80°C before analysis. Dopamine extraction, acylation, and enzyme immunoassay were performed according to the manufacturers' instructions. Every sample and standard was performed in duplicated. Absorbance was read using a microplate reader set to 450 nm and a reference wavelength set between 620 and 650 nm. Data were from four independent experiments.

### Statistical analysis

Data were expressed as the mean ± SD. SPSS 16.0 was used to perform the statistical analysis. One-factor ANOVA was used to assess statistical significance between the means. The statistical significance was established at *P* < 0.05 (^*^p < 0.05, ^**^p < 0.01).

## Results

### Our new defined culture condition significantly increased the expression of DA neuron-associated genes in the differentiated forebrain-derived hNSCs

**Figure 1:**
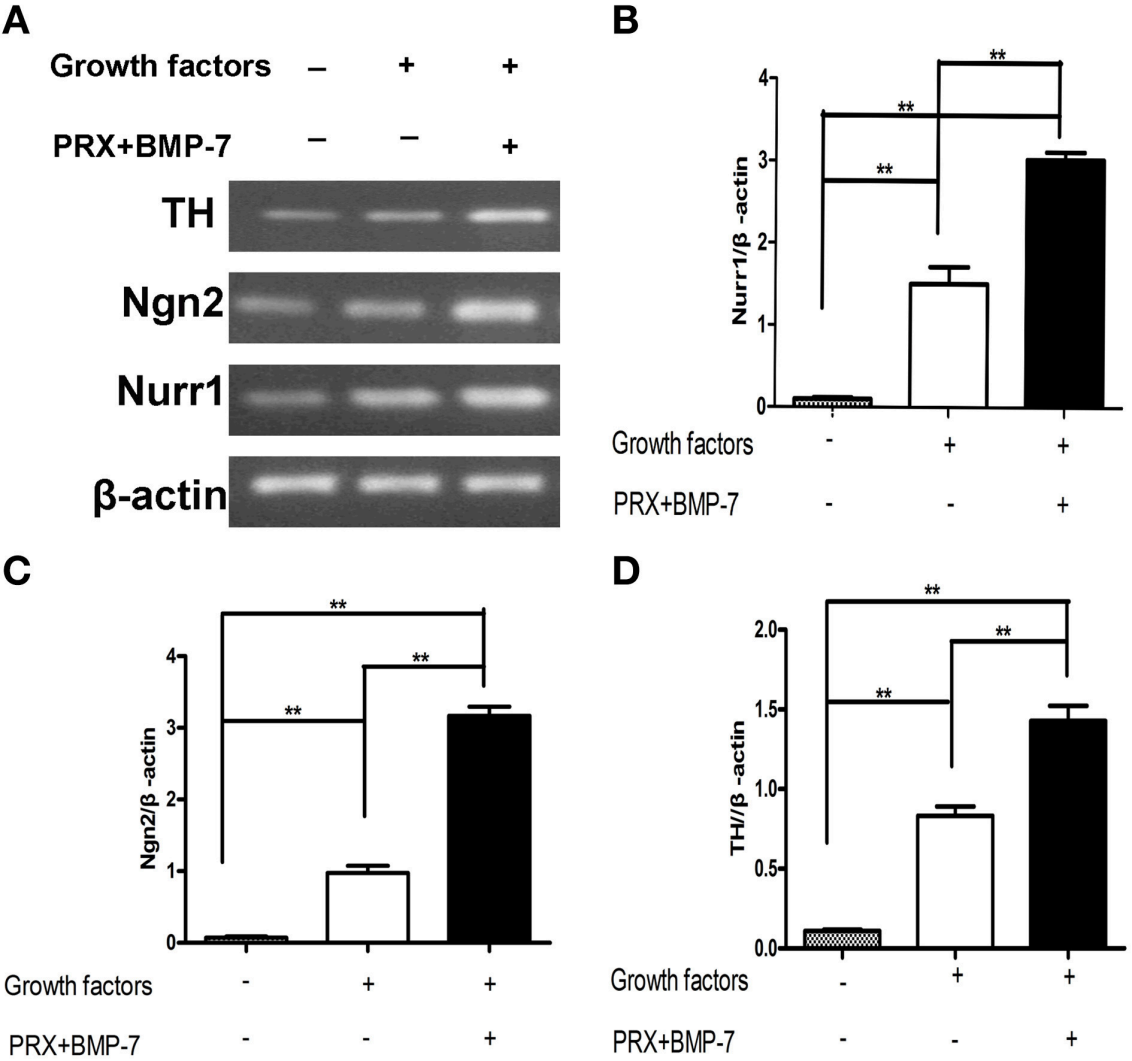
**The combined application of BMP-7, pramipexole, and growth factors in culture significantly increased the expression of DA neuron-associated genes of forebrain-derived hNSCs. (A)** Gene expression of Nurr1, Ngn2, and TH examined by RT-PCR at 24 h of differentiation. Forebrain-derived hNSCs were treated with 1% FBS, 1% FBS and growth factors, and 1% FBS, BMP-7, pramipexole, and growth factors, respectively. **(B–D)** Bar graphs showing that the gene expression of Nurr1, Ngn2, and TH was significantly improved by the combined application of BMP-7, pramipexole, and growth factors in cultures compared to controls. Human β-actin gene fragment was used for internal control. All experiments were performed with four independent cDNAs. PCR products were analyzed by electrophoresis in 1.5% agarose gel containing ethidium bromide and quantified by densitometric analysis with quantity one system. Data were presented as mean ± S.E.M. One-factor ANOVA was used to assess statistical significance between means (^**^P < 0.01).

To examine whether our new defined culture condition has any biological effect on DA neuron-associated genes, RT-PCR was performed to measure the expression of Nurr1, Ngn2, and TH genes in the forebrain-derived hNSCs at 24 h after *in vitro* differentiation. As illustrated in Figures 1A–D, the expression of these DA neuron-associated genes were significantly augmented under our new defined culture condition compared to control groups (*p* < 0.01).

### TH-, VMAT2-, and DAT-positive cells were significantly increased under our new defined culture condition

**Figure 2:**
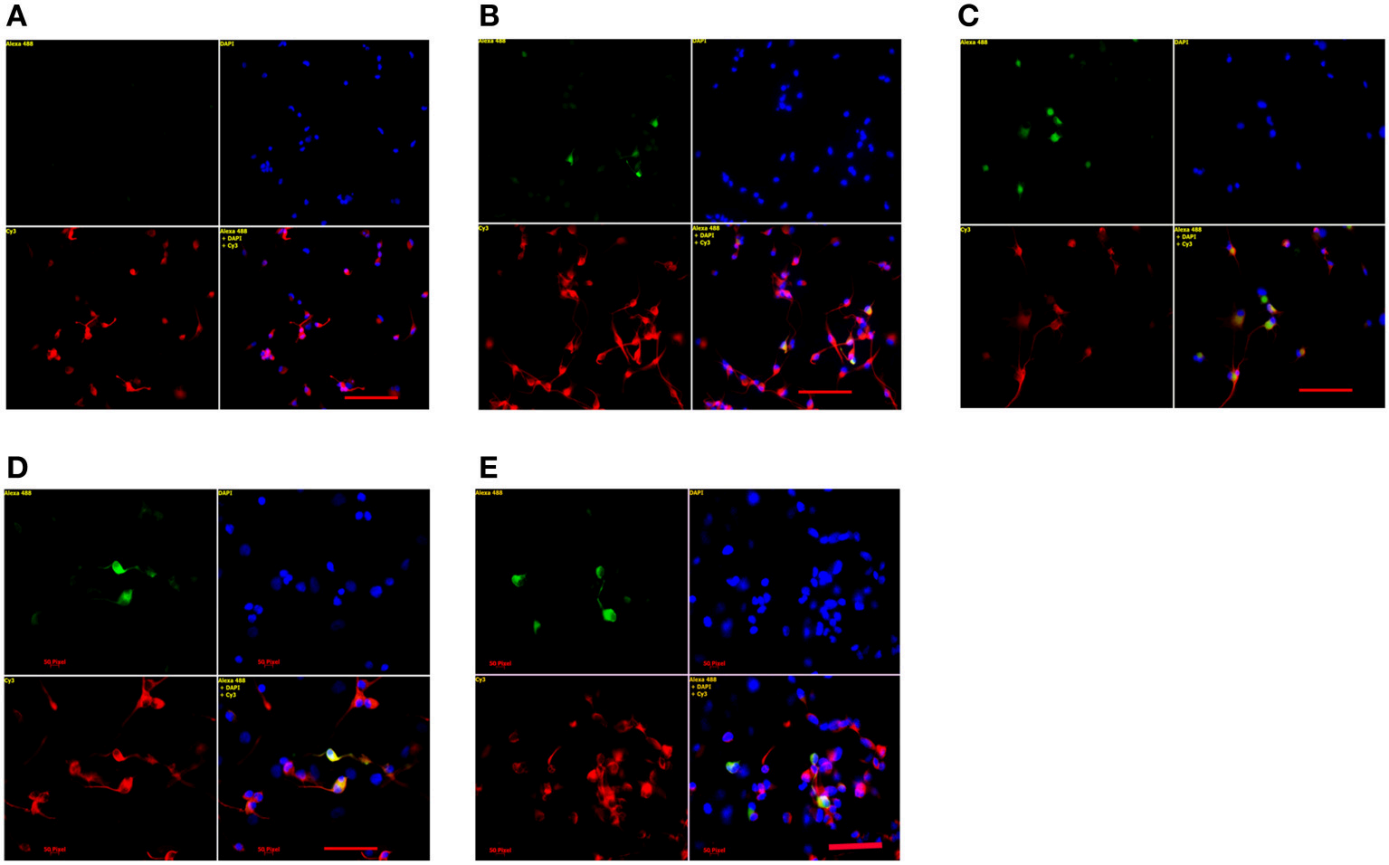
**TH-, VMAT2-, and DAT-positive cells were detected in the cultures with the combined application of BMP-7, pramipexole, and growth factors. (A–C)** TH-positive neurons in **(A)** 1% FBS, **(B)** 1% FBS and growth factors, and **(C)**: 1% FBS, BMP-7, pramipexole, and growth factors examined by fluorescent immunocytochemistry examined at 24 h of differentiation. Green: TH-positive cells, Red: β-tubulin III-positive cells, Blue: DAPI labeled nuclei, Yellow: co-localization of TH (green) and β-tubulin III (red) in the same cells. Bar = 200 μm. **(D)** VMAT2-positive neurons in cultures with 1% FBS, BMP-7, pramipexole, and growth factors examined at 72 h of differentiation by fluorescent immunocytochemistry. Green: VMAT-2-positive cells, Red: β-tubulin III-positive cells, Blue: DAPI labeled nuclei, Yellow: co-localization of TH (green) and β-tubulin III (red) in the same cells. No VMAT2-positive neurons were detected in cultures of the controls. Bar = 100 μm. **(E)** DAT-positive neurons in cultures with 1% FBS, BMP-7, pramipexole, and growth factors examined at 72 h of differentiation by fluorescent immunocytochemistry. Green: DAT-positive cells, Red: β-tubulin III-positive cells, Blue: DAPI labeled nuclei, Yellow: co-localization of DAT (green) and β-tubulin III (red) in the same cells. No DAT-positive neurons were detected in cultures of the controls. Bar = 100 μm.

**Figure 3:**
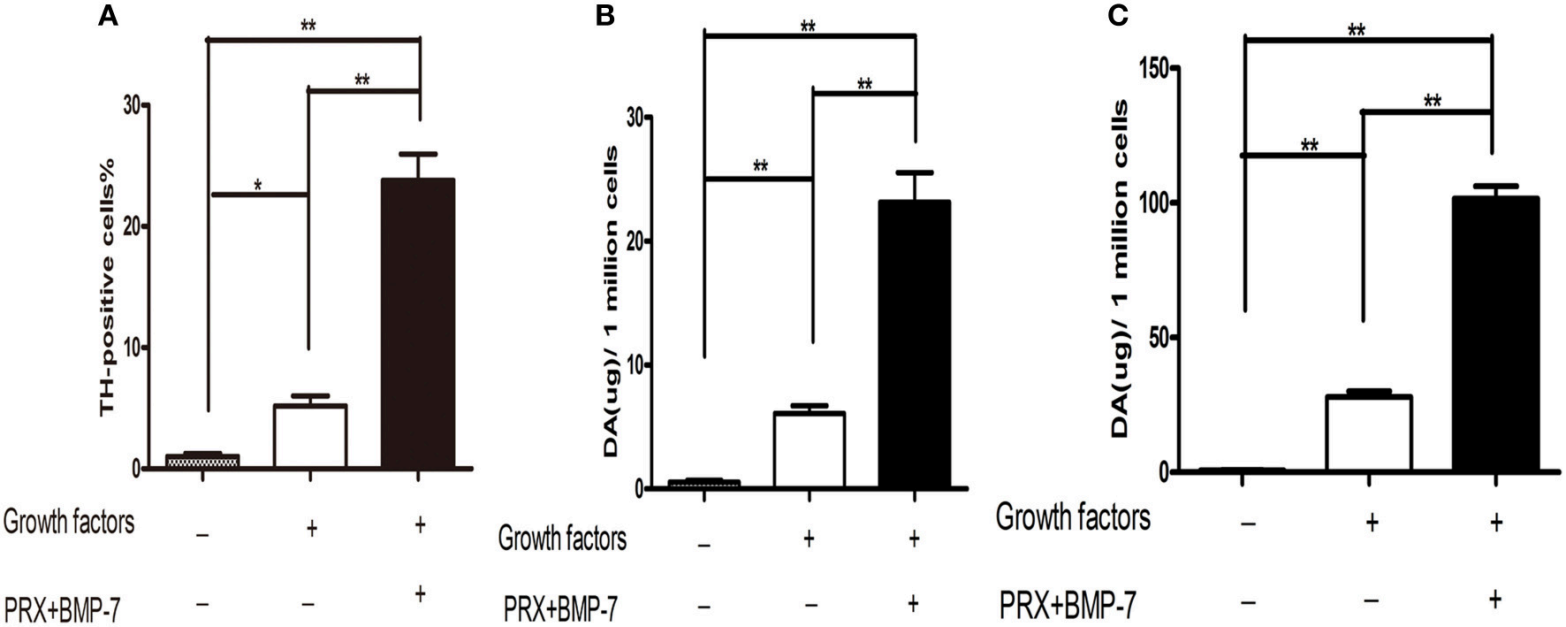
**Dopamine release was significantly increased at both basal and evoked levels in DA neurons produced in cultures containing 1% FBS, BMP-7, pramipexole, and growth factors. (A)** Bar graphs showing that the number of DA neurons produced in cultures containing 1% FBS, BMP-7, pramipexole, and growth factors were significantly increased compared to controls. **(B,C)** The basal **(B)** and evoked **(C)** levels of dopamine released by DA neurons were significantly augmented in cultures containing 1% FBS, BMP-7, pramipexole, and growth factors compared to those in cultures of the controls. Data were received with four independent experiments. Basal or evoked dopamine releases were assessed by ELISA. Data were presented as mean ± S.E.M. One-factor ANOVA was used to assess statistical significance between means (^**^P < 0.01, ^*^P < 0.05).

After 24 and 72 h of differentiation, fluorescent immunocytochemistry was conducted to examine TH-, DAT-, and VMAT2-positive neurons in differentiated forebrain hNSCs. As shown in Figures 2A–C, 3A, our defined culture condition significantly increased the number of TH-positive cells in the culture after 24 h of differentiation compared to control groups. In addition, these TH-positive neurons possessed longer neuronal processes or fibers than those in control groups (Figures 2A–C). However, VMAT2- and DAT-positive cells were not detected at 24 h until at 72 h after differentiation using our new defined culture condition. VMAT2-positive cells occupied about 5%, and DAT-positive cells occupied less than 5% of the total population of the neuronal cells in the culture (Figures 2D,E). Most of the TH-positive cells, DAT-positive cells, and VMAT2-positive cells co-expressed β-tubulin III, the marker for neurons. In control groups, no DAT- or VMAT2-positive cells were detected at either 24 or 72 h of differentiation.

### The levels of dopamine release by the differentiated DA neurons were significantly increased at basal and evoked conditions in our new defined culture system

To confirm whether the differentiated DA neurons from forebrain hNSCs are functional and release dopamines in cultures, the basal, and evoked levels of dopamine releases by these cells were respectively assessed with ELISA. As shown in Figures 3B,C, not only the basal levels but also the evoked levels of dopamine release were significantly increased compared to those in control groups. Thus, these DA neurons differentiated from forebrain-derived hNSCs in our defined culture system were DA-producing neurons and may serve as suitable cell sources for PD treatment.

## Discussion

hNSCs derived from forebrain can be expanded for long time as free-floating neurospheres and differentiated into neurons, astrocytes and oligodendrocytes. Thus, we can get plenty of hNSCs by *in vitro* culture without losing their multipentcy. However, the low efficiency to generate functional dopamine neurons from forebrain-derived hNSCs limited their therapeutic potential as the donor cells. In the current study, we used a new culture condition, supplemented with PRX, BMP-7, and growth factors, to differentiate more DA neurons from the forebrain-derived hNSCs. We generated DA neurons with the expression of both TH and VMAT2, and these DA neurons were confirmed to release dopamine in cultures.

PRX is known to induce the expression of Nurr1 in SH-SY5Y cells. BMP-7 is confirmed to not only be involved in the expression of Ngn2 in mouse cortex neurons but also maintain the steady-state levels of Ngn2 transcripts in the developmental window between E14-15 (Segklia et al., 2012). As expected, both gene expression of Nurr1 and Ngn2 in differentiated hNSCs of forebrain origin were induced and augmented significantly using our defined culture condition compared to those in control groups (Figures 1A–C). Nurr1, an orphan nuclear receptor, is crucial for DA differentiation and maintenance, usually expressed in post-mitotic DA neuroblasts prior to TH expression. The expression of TH was also successfully induced after 24 h of differentiation under our new defined culture condition (Figure 1A). Our results suggest that the expression of Nurr1 may climb the steady peak before 24 h of differentiation, the Ngn2 may promote hNSCs to differentiate toward mature neurons, and over-expression of Nurr1 and Ngn2 in combination may generate morphologically mature TH- expressing neurons (Andersson et al., 2007). Consistent with the experimental data obtained from mesencephalic progenitors, TH- expressing neurons produced under our new defined culture condition have longer neuronal processes and fibers (Figures 2A–C).

TH is the enzyme that catalyzes the initial, rate-limiting step in the biosynthesis of catecholamines, including DA, noradrenaline, and adrenaline. Expression of TH alone is not sufficient to prove that the neurons are dopaminergic. VMAT2 is responsible for packaging newly synthesized or recaptured DA into the synaptic vesicles, and therefore controls the concentration and disposition of cytoplasmic DA within the nerve terminal. Thus, VMAT2 is a marker for mature and functional DA neurons. We did not find VMAT2-positive neurons at 24 h of differentiation but at 72 h of differentiation using our new defined culture condition (Figure 2D). DAT, as another marker for mature and functional DA neurons, plays a key role in terminating dopaminergic signaling and in maintaining a releasable pool of dopamine (German et al., 2015). DAT-positive neurons at 24 h of differentiation were not detected until at 72 h of differentiation by using our culture condition (Figure 2E). These data suggest that our new defined culture condition may have efficiently generated mature and functional DA neurons from forebrain-derived hNSCs. However, further studies are needed to assess the efficiency of the proposed protocol for long term differentiation of DA neurons, since the degree of DA differentiation showed after 72 h is quite limited.

The most important physiological aspect of functional DA neurons is the ability to synthesize and release DA neurotransmitter in response to membrane depolarization. In the current studies, ELISA was used to test the basal (DA neurotransmitter at resting status) and evoked (DA neurotransmitter release by responding to cell membrane depolarization) DA neurotransmitter release. As shown in Figures 3B,C, DA neurons produced by our protocol not only have the capability to synthesize DA but also release DA in response to membrane depolarization evoked by KCl than those obtained in control groups. Collectively, our results showed that the combined application of PRX and BMP-7 with growth factors in culture greatly promote the production of more DA-releasing neurons from forebrain-derived hNSCs.

## Author contributions

HY peformed the experiment and wrote the manuscript. JW performed the PCR, ELISA and cell culture. FW, XL, and HC performed cell culture, cell differentiation, and immunocytochemistry. WD helped to designed the experiment. TQ designed the whole experiment and revised the manuscript.

## Fundings

This research is supported by grants of (1) DoD (PR100499P1), (2) The Boothroyd Foundation, USA, (3) 5150 Program of Jinan City, and (4) Taishan Oversea Scholars Program of Shandong Province, China to TQ.

### Conflict of interest statement

The authors declare that the research was conducted in the absence of any commercial or financial relationships that could be construed as a potential conflict of interest.
